# Novel classification for simple peripheral arteriovenous malformations based on anatomic localization: Prevalence data from the tertiary referral center in China

**DOI:** 10.3389/fcvm.2022.935313

**Published:** 2022-07-22

**Authors:** Yuchen Shen, Zhenfeng Wang, Xitao Yang, Lianzhou Zheng, Mingzhe Wen, Yifeng Han, Xiao Li, Liming Zhang, Jingbing Wang, Jianxiong You, Chunyu Jiang, Lixin Su, Xindong Fan, Deming Wang

**Affiliations:** Vascular Anomaly Center, Department of Interventional Therapy, Shanghai Ninth People's Hospital, Shanghai Jiao Tong University School of Medicine, Shanghai, China

**Keywords:** arteriovenous malformation, prevalence, classification, anatomy, diagnosis

## Abstract

**Background:**

In absence of the large-sample study of simple peripheral arteriovenous malfomations (pAVM), we aimed to perform the epidemiological analysis of over 1,000 simple pAVM patients from our center in the past 5 years, and establish a novel classification based on the anatomical localization of the primary lesion.

**Results:**

Between March 27, 2016, and March 31, 2021, Chinese patients who were diagnosed with simple pAVM were taken into account. Those who suffered from simple arteriovenous malformations of the central nervous system (cnsAVM), combined types of AVM, and syndromes, such as CLOVES syndrome, etc. were all excluded from this study. A total of 1,070 simple pAVM patients were screened out. All of the simple pAVM patients were diagnosed by clinical manifestations and imaging examinations. Demographic data were obtained from the National Bureau of Statistics of China. The 5-year prevalence of simple pAVM was about (2.15–6.60) /1,000,000 population. The male-female ratio was approximately 1.22:1. The pAVM inpatients that were included in the age group of 21~30 years old had the highest constituent ratio (*P* = 0.01). The classification included four groups: Type I (primarily occurring in soft tissue); Type II (primarily occurring in bone); Type III (primarily occurring in the viscus) and Type IV (simple pAVM coexisting with CNS lesions). There were two subtypes of Type I: the A subtype (involving one major anatomical region) and the B subtype (involving two or more major anatomical regions); two subtypes of Type II: the A subtype (the cortex was intact) and the B subtype (the lesion had broken through the cortex). Generally, 657 patients were classified as Type IA (61.4%), 232 patients were Type IB (21.7%), 82 patients were Type IIA (7.7%) and 79 were categorized as Type IIB (7.4%); the number of patients who had Type III and Type IV pAVM were 9 (0.8%) and 11 (1.0%), respectively. The clinical manifestations and diagnostic standards for each type were also systematically summarized.

**Conclusions:**

Prevalence data for simple pAVM were analyzed, and a novel classification was proposed based on the anatomy of the lesions. The present work was expected to facilitate the diagnosis of simple pAVM in clinical works.

## Introduction

Arteriovenous malformations (AVM) have a low incidence, however, they always pose a difficult situation to clinicians and decrease the AVM patients' quality of life (1).

AVMs of the central nervous system (cnsAVM) are inclined to cause severe neurological deficits resulting from hemorrhage, spinal venous congestion, or cord compression (2). In contrast, because of the insidious onset during patients' growth and the fact that peripheral arteriovenous malformations (pAVM) usually do not cause fatal consequences at an early stage, the presence of a pAVM was prone to be ignored by either patients or clinicians and always progresses to a more extensive status when they were diagnosed. The clinical manifestation and natural course of pAVM are closely related to its primary region (3). For instance, pAVM in the head and neck region are likely to destroy a patient's facial appearance, which can also leave irreversible psychological problems (4). For pAVM in the extremities, the existence of the “steal phenomenon” leads to the occurrence of ischemia, ulcers and necrosis in the lesion location. Sometimes, an amputation is required in severe cases (5). As a large amount of arterial blood directly flows into the venous system and eventually backflows into the right atrium and right ventricle, pAVM of the major named vessels in the trunk are likely to cause congestive heart failure symptoms and impose constraints on the patient's daily life (6).

Nowadays, the widely used classification for pAVM was based on the angiographic characteristics of the “nidus” (7), which means the angiographic classification cannot be applied until invasive angiography is performed. Besides, the angiographic classification only focused on the localized morphology of vascular disorders, lacking of the holistic description of the lesion. Given the diversity of clinical manifestations and locations of primary lesion, obstacles and confusions still exist in the pre-operation diagnosis of pAVM (8). Hence, there is an urgent need to propose the classification that can effectively distinguish the clinical characteristics of pAVM.

According to the International Society for the Study of Vascular Anomalies (ISSVA) up-to-date classification of vascular malformations, pAVM could be divided into simple and combined types (9). As the major tertiary referral center specializing in pAVM in China, totally 1,106 first-visit patients with AVM were hospitalized and received treatments in the past 5 years. Here, we retrospectively analyzed the clinical data, and put forward our novel classification of simple pAVM based on the anatomical regions and the nature of the primary lesion, aimed to provide a more reliable reference for clinicians in preoperative diagnosis of simple pAVM.

## Materials and methods

### Acquisition of patient data and demographics

This study was approved by the Human Research Ethics Committee of the Shanghai Ninth People's Hospital, Shanghai Jiao Tong University School of Medicine (Shanghai, China). Given the retrospective nature of this study, the need for informed consent was waived. Approval was also obtained from the institutional review board of the Shanghai Ninth People's Hospital for retrospective review of the patients' medical and imaging records.

Demographic data from March 2016-March 2020 were obtained from the National Bureau of Statistics of China (https://data.stats.gov.cn/).

### Patients

Inpatients at the Department of Interventional Therapy, Shanghai Ninth People's Hospital, Shanghai Jiao Tong University School of Medicine, were hospitalized between March 27, 2016, and March 31, 2021. In total, 2,397 AVM cases and 1,106 first-visit AVM patients were recorded. The diagnosis of AVM was established by the clinical manifestations and imaging examinations. The clinical manifestations varied according to the disparate lesion locations and the different AVM stages. Imaging examinations, including magnetic resonance imaging (MRI) contrast-enhanced computed tomography (CT), and digital subtraction angiography (DSA), were performed during the operations.

### Patients' inclusion and exclusion criteria

The inclusion criteria:

1) Patients came from China.
2) Patients who were diagnosed with simple pAVM.

The exclusion criteria:

1) Patients with simple AVM only occurred in the central nervous system (brain and spinal cord).
2) Patients with the combined types of AVM (capillary-arteriovenous malformation, capillary-lymphatic-arteriovenous malformation, capillary-venous-arteriovenous malformation, and capillary-lymphatic-venous-arteriovenous malformation, etc.).
3) Patients' AVM was one of the phenotypes of the syndromes (CLOVES syndrome, etc.).

### Establishment methods of the pAVM anatomical classifications

According to the putative reference of the systematic anatomy, each part of the human body was divided into major anatomical regions (MARs) (10, 11):

1) Head and neck: neck, mentum, lower lip, oral cavity, upper lip, pharyngeal, buccal region, maxillary region, nasal region, orbital region, zygomatic region, parotideomasseteric region, deep region of the lateral face, frontal part, temporal region, ears, scalp.
2) Trunk: thorax, abdomen, back.
3) Upper limbs: shoulder, upper arm, elbow, forearm, hand.
4) Lower limbs: gluteal region, thigh, knee, popliteal space, shank, foot.

### Statistical analysis

Normality of data was tested by the D'Agostino & Pearson test. Normally distributed data are depicted as the mean ± standard deviation (SD), while non-normally distributed data are depicted by the median and interquartile range (IQR). Two-sided chi-squared test was used to analyze the correlation between sex and simple pAVM occurrence. Two-sided one-sample *t*-tests were used to analyze the significance of the differences within the cohorts. The statistical analysis was processed by Prism GraphPad Prism 8 (GraphPad Software, San Diego, CA, USA). Adobe Photoshop CS6 (San Jose, CA, USA) was applied for graphical production.

## Results

### Epidemiological data of simple pAVM

#### Geographical distribution and prevalence

**Figure 1:**
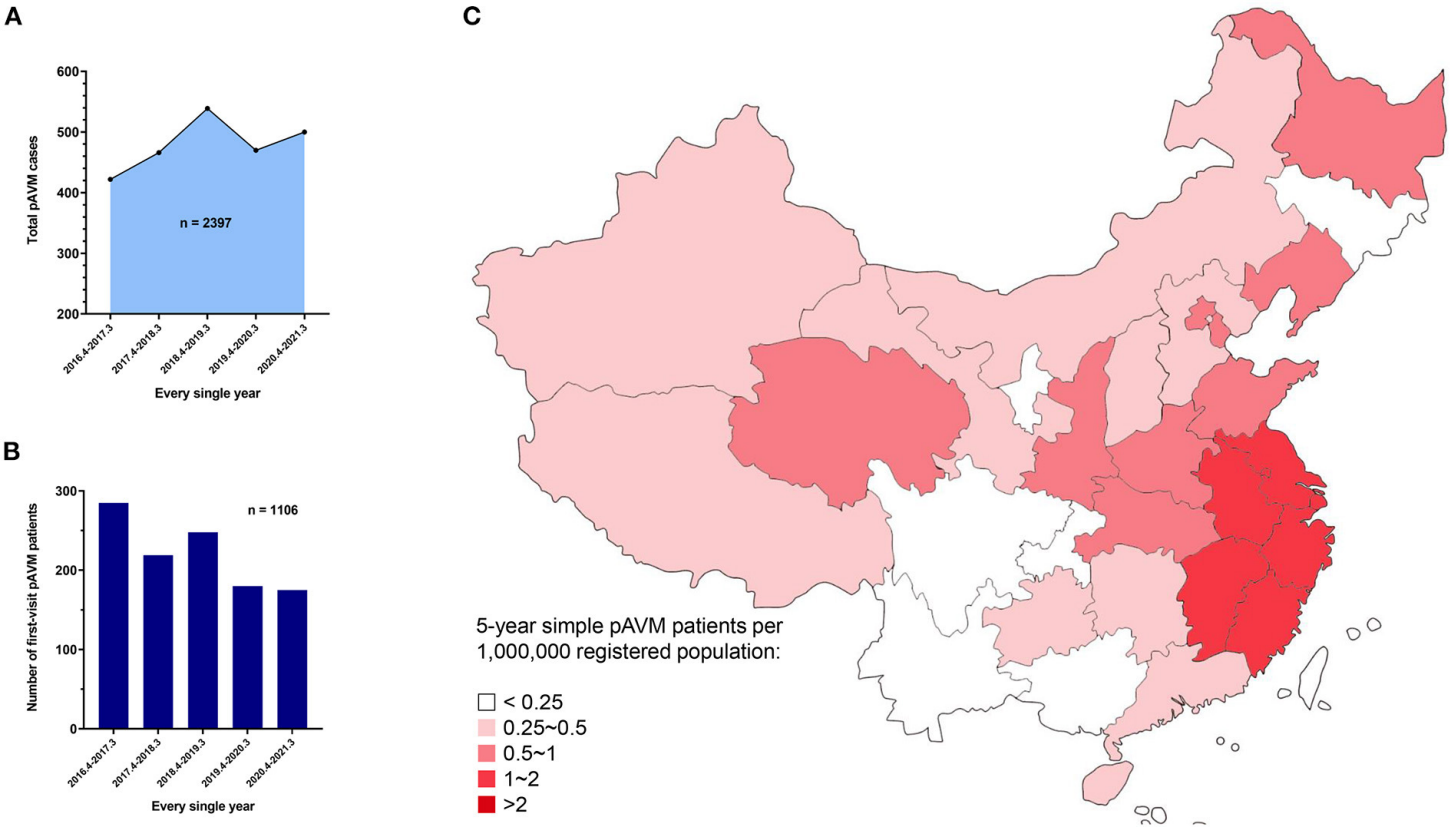
Overall 5 year AVM and simple pAVM data. **(A)** AVM cases in every single year from March 27th, 2016 to March 31st, 2021 (*P* < 0.0001). **(B)** First-visit AVM patients in every single year from March 27th, 2016 to March 31st, 2021 (*P* = 0.0004). **(C)** Heat map of the distribution of simple pAVM patients in China.

During the past 5 years, we have received pAVM patients with a relatively stable number of patients presenting for every single year (Figures 1A,B). A heatmap based on the registered population of each province was generated to demonstrate the distribution of pAVM patients intuitively (Figure 1C). Furthermore, the specific number of patients in each province was shown in Supplementary Figure 1.

**Figure 2:**
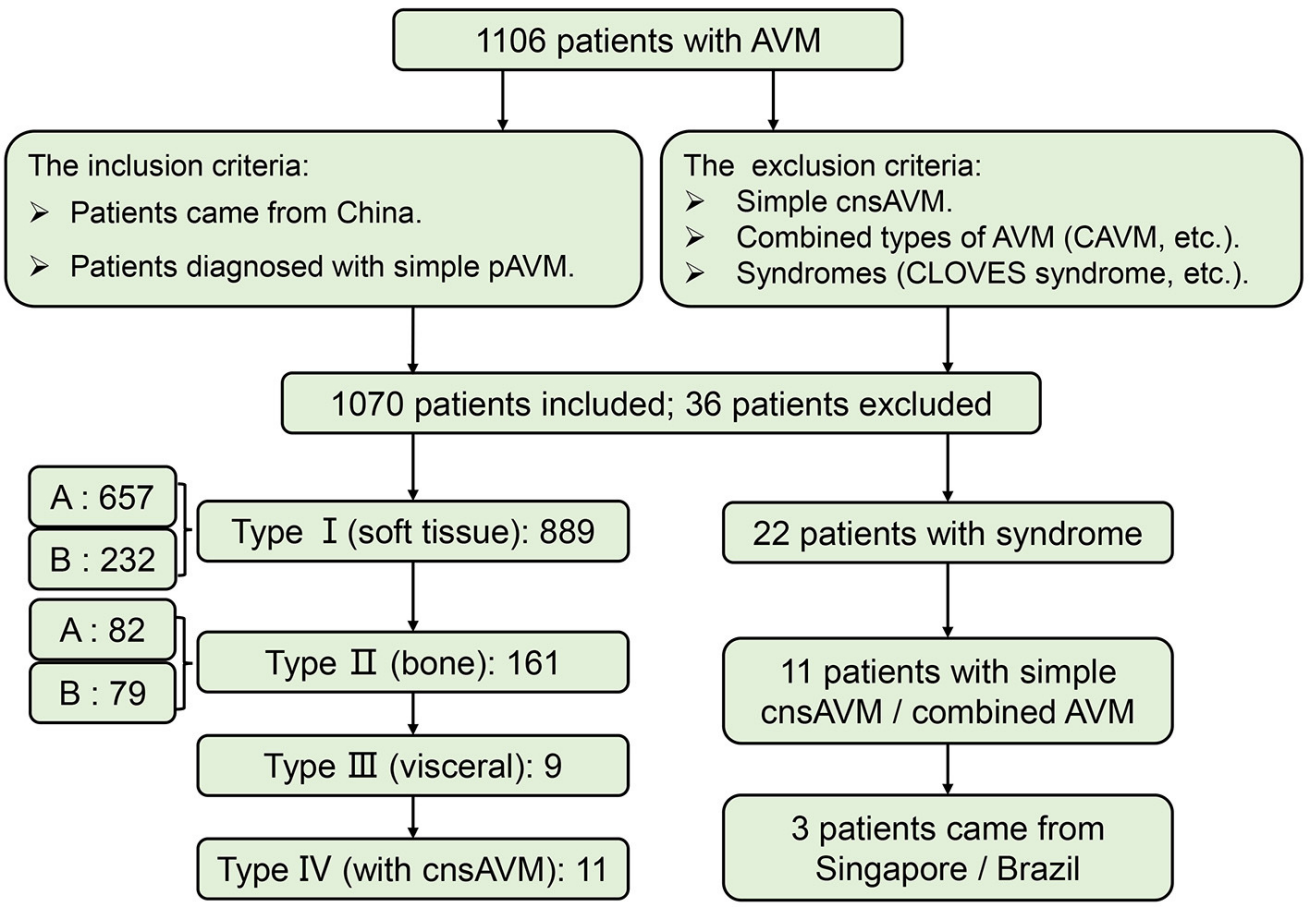
Flow of participants in the study. CAVM, capillary-arteriovenous malformation.

Only Chinese patients who were diagnosed with simple pAVM were taken into account. Those who suffered from simple cnsAVM, combined types of AVM which was defined by the ISSVA (9), and syndromes, such as CLOVES syndrome (congenital lipomatous, overgrowth, vascular malformations, epidermal nevi, scoliosis/skeletal and spinal syndrome), etc. were all excluded from this study. Finally, 1,070 simple pAVM patients were screened out for subsequent analyses (Figure 2). The official demographic data of China from 2016 to 2020 was obtained. As the major center, we received and treated almost all of the pAVM patients in Shanghai; thus, we simply calculated the prevalence based on the statistical data of Shanghai. The 5 year average registered population of Shanghai was 14,690,000, the number of 5 year simple pAVM patients was 97, and the 5 year prevalence of simple pAVM was 6.60 (per a population size of 1,000,000). Second, in addition to Shanghai, we selected three other nearest provinces: Anhui, Jiangsu, Zhejiang. The 5 year average registered population of these four regions was 214,858,500, the number of 5 year simple pAVM patients was 463, and the 5 year prevalence of simple pAVM was 2.15 (per a population size of 1,000,000).

#### Sex and age

**Figure 3:**
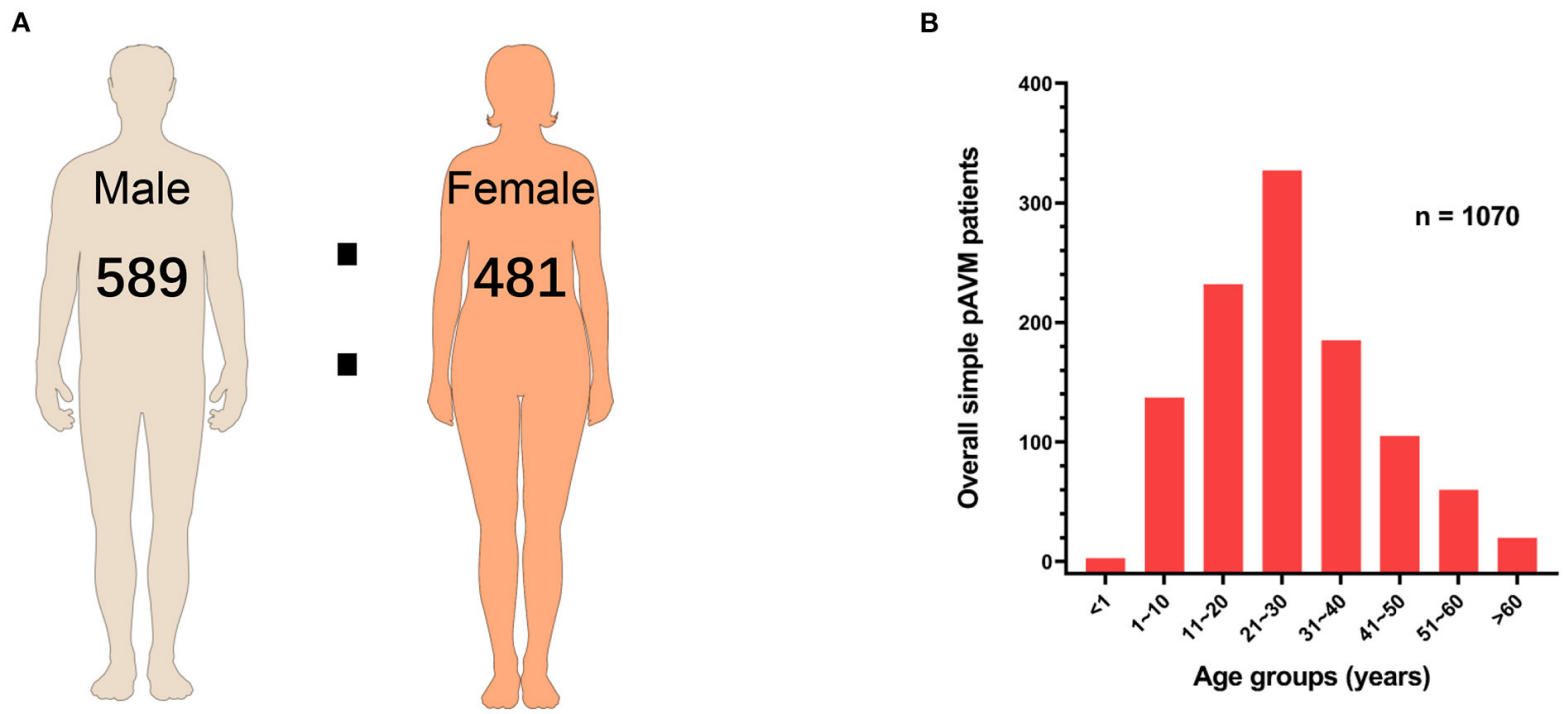
Sex and age of simple pAVM patients. **(A)** Sex ratio of simple pAVM. **(B)** Age distribution of simple pAVM (*P* = 0.01).

To exclude the interference of sex distribution in the general population on the sex distribution of pAVM patients, a chi-squared test was performed. As Table 1 showed, sex was the influence factor of pAVM occurrence (*P* = 0.013). Among 1,070 simple pAVM patients, 589 were male and 482 were female, and the male-female ratio was approximately 1.22:1 (Figure 3A). Participants were aged from 0.5 to 83 years old with the IQR: 16–35 years old (median: 25.5 years old). The simple pAVM patients who were in the age group 21~30 years had the highest constituent ratio. Interestingly, for those who were under 20 years of age, the number rose steeply by each decade; however, for those over 30 years, the simple pAVM constituent ratio dropped sharply in the first decade but tended to be flat in subsequent decades (Figure 3B).

**Table 1:** The correlation between sex and simple pAVM occurrence.

**Sex**	**Simple pAVM^a^**	**Total population^b^**	*χ^2^*	***P*** **-value^c^**
Male	589 (55.0%)	723,570,000 (51.2%)	6.21	0.013^*^
Female	481 (45.0%)	688,550,000 (48.8%)		

a pAVM, peripheral arteriovenous malformations.

b Based on the data from the National Bureau of Statistics of China.

c Statistical test was 2-tailed.

* , P < 0.05.

### The anatomical classification for simple pAVM

#### Definition of the anatomical classification system

The anatomical classification system is presented in Table 2.

**Table 2:** Anatomical classification for simple pAVM.

Type I *AVM^a^ primarily occurred in soft tissue*.	A: AVM involving one MAR^c^.
	B: AVM involving two or more MARs.
Type II *AVM primarily occurred in bone*.	A: AVM did not break through the cortex (intraosseous AVM).
	B: AVM broke through the cortex (involving soft tissue).
Type III *AVM primarily occurred in viscus*.	Visceral AVM.
Type IV *PAVM^b^ coexisted with cnsAVM*.	PAVM and cnsAVM were mutually independent or interconnected.

a AVM, arteriovenous malformations.

b pAVM, peripheral arteriovenous malformations.

c MAR, main anatomical region.

#### The overall distribution of each subtype

**Figure 4:**
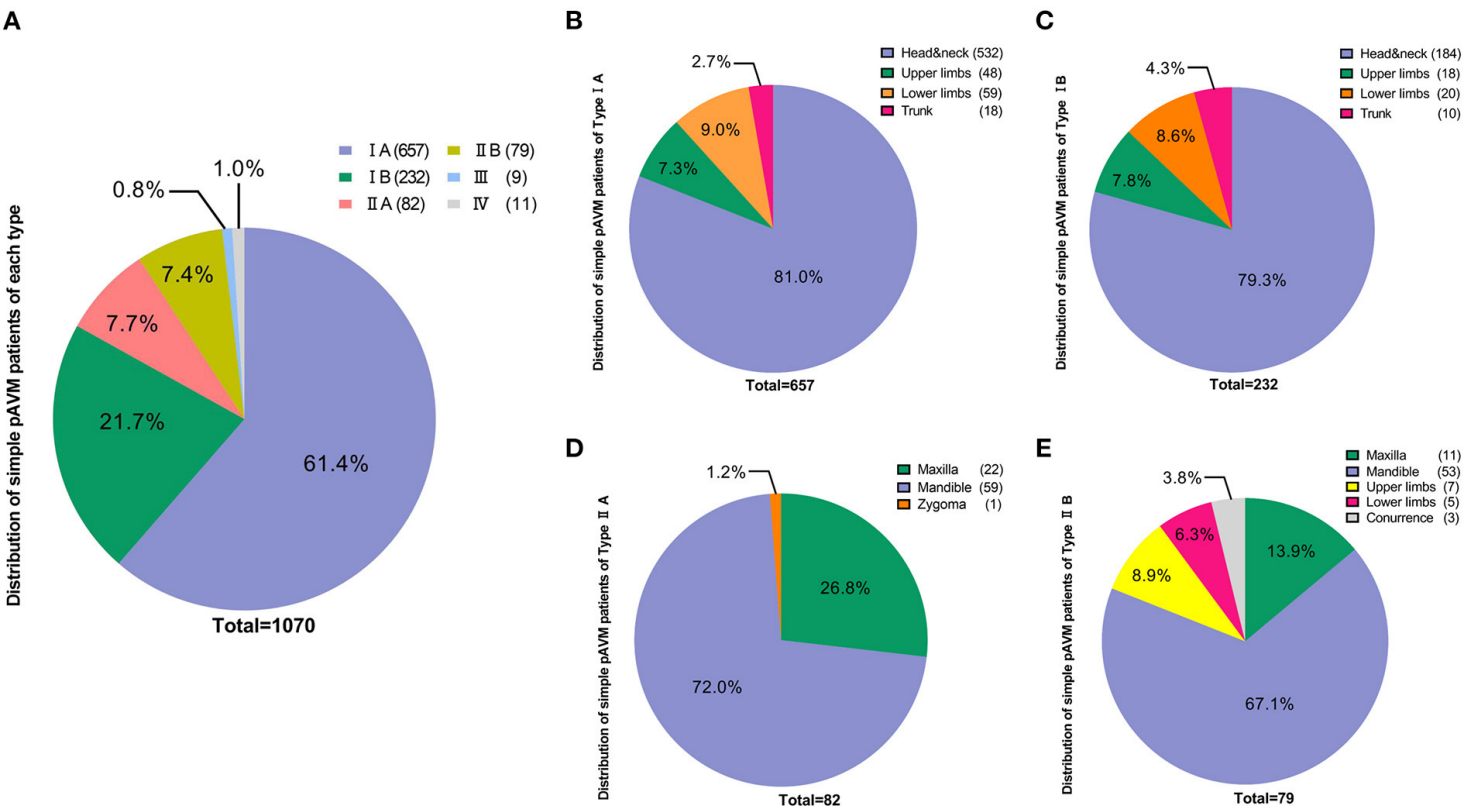
Distribution of simple pAVM cohort according to classification. **(A)** Number of simple pAVM patients of each subtype. **(B)** Number of Type IA simple pAVM patients of each location. **(C)** Number of Type IB simple pAVM patients of each location. **(D)** Number of Type IIA simple pAVM patients of each location. **(E)** Number of Type IIB simple pAVM patients of each location (Concurrence: patients with simple pAVM in both maxilla and mandible).

According to our classification, 657 patients were classified as Type IA (61.4%), 232 patients were Type IB (21.7%), 82 patients were Type IIA (7.7%) and 79 were categorized as Type IIB (7.4%), 9 patients had type III (0.8%) and 11 patients had type IV simple pAVM (1.0%) (Figure 4A). We first performed statistics on the location of lesions of subtypes I and II. For type IA and IB simple pAVM, patients with lesions in the head and neck region had the highest constituent ratios (532 and 184) and patients who had lesions in the trunk had the lowest constituent ratios (18 and 10). Given a large number of patients and the anatomical complexity, a statistical analysis was performed exclusively for Type IA simple pAVM of the head and neck region (Table 3). The number of Type IA patients was relatively larger than that of Type IB in each region (Figures 4B,C), and for Type II simple pAVM, the head and neck was still the region with the highest constituent ratio (Figures 4D,E). Moreover, the mandibular AVMs were more common than the maxillary AVMs (112 and 33). It should be noted that only Type IIB, but not IIA simple pAVM of the limbs were observed in our cohort, suggesting that patients have always perceived the lesion after the soft tissues were involved.

**Table 3:** Distribution of 532 Type I A simple pAVM of head and neck region based on MARs.

**Anatomic regions^a^**	**Number of cases (%)**
Neck	22 (4.2%)
Mentum	9 (1.7%)
Lower lip	38 (7.3%)
Oral cavity	32 (6.0%)
*Floor of mouth*	3
*Tongue*	24
*Gingiva*	1
*Palate (hard and soft palate)*	4
Upper lip	49 (9.2%)
Pharyngeal	1 (0.2%)
Buccal region	62 (11.6%)
Maxillary region	77 (14.4%)
Nasal region	27 (5.1%)
Orbital region	50 (9.4%)
Zygomatic region	8 (1.5%)
Parotideomasseteric region	12 (2.6%)
Deep region of lateral face	2 (0.4%)
Frontal part	28 (5.2%)
Temporal region	13 (2.4%)
Ears	75 (13.9%)
Scalp	27 (5.1%)

a *According to Human Anatomy, Head and Neck Chapter*.

### Clinical manifestation for each type

#### Type I simple pAVM (soft tissue)

**Figure 5:**
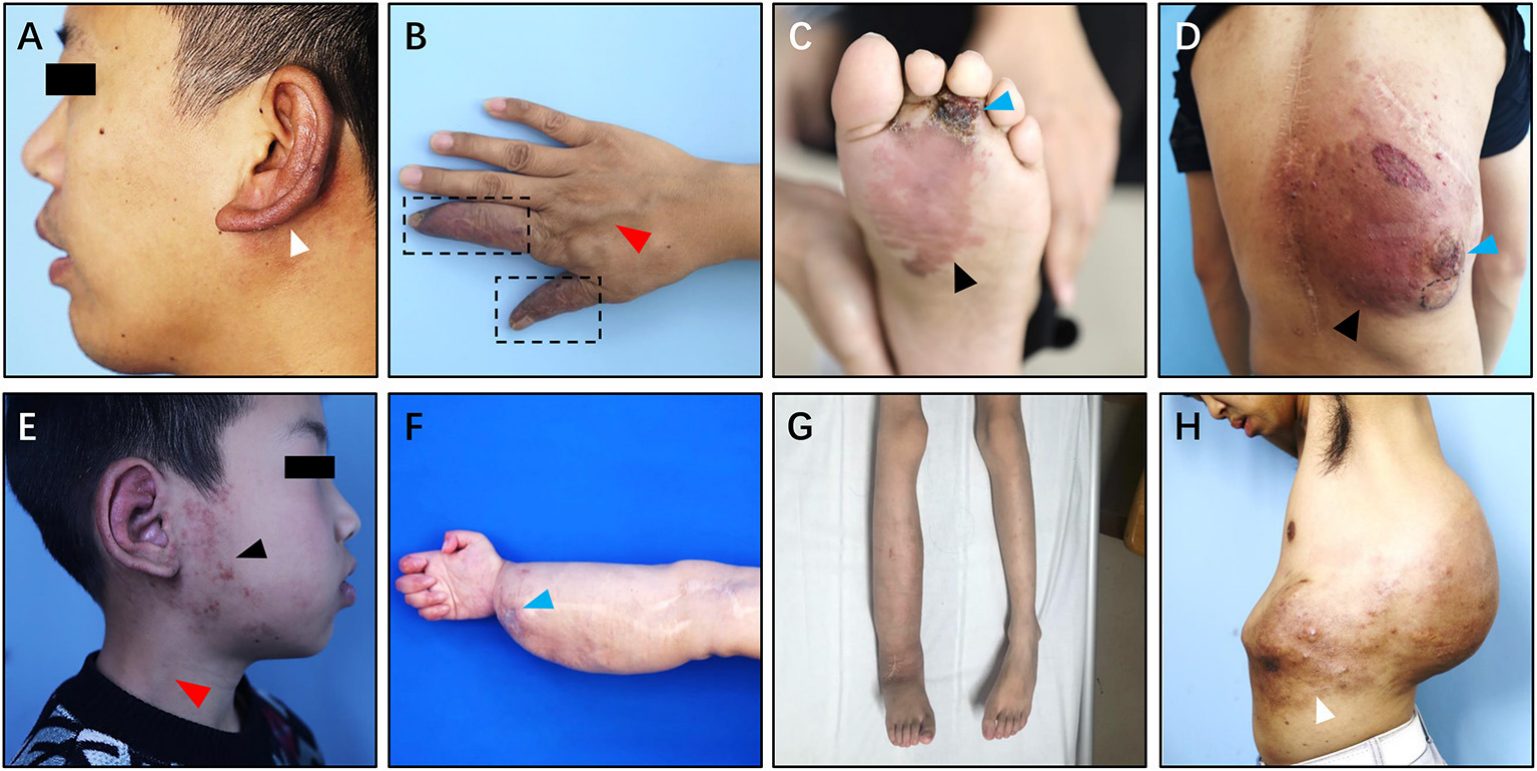
Clinical manifestation of Type I simple pAVM. **(A)** Patient 1 with auricular AVM (type IA). **(B)** Patient 2 with AVM of hands (type IA). **(C)** Patient 3 with AVM of left foot (type IA). **(D)** Patient 4 with AVM of back (type IA). **(E)** Patient 5 with AVM of ear and parotideomasseteric region (type IB). **(F)** Patient 6 with AVM of right hand, forearm and elbow (type IB). **(G)** Patient 7 with AVM of trunk and gluteal region (type IB). **(H)** Patient 8 with AVM of back and abdomen (type IB). White arrow: cutaneous chromatosis. Dark arrow: cutaneous erythema. Red arrow: dilation of the outflow vein or external jugular vein. Blue arrow: scab or ulceration. Dark dotted box: the “steal phenomenon.”

The common clinical symptoms of Type I simple pAVM include cutaneous erythema, a soft tissue mass or swelling, and superficial venous dilation, and patients may feel pain in some circumstances (Figure 5) (12). The physical examinations may show that the lesion is a dark red or pink color, there is a poorly defined soft tissue distention and there are dilated capillaries (12). Furthermore, the involved area may show skin discoloration accompanied by hyperthermia, persistent pulsation, and blowing bruits, tinnitus was especially common in patients with auricular AVM (Figures 5A,E) (13). Tortuousness and dilation of outflowing veins can be seen in Type I simple pAVM (Figure 5B). Particularly, in patients with AVMs of the head and neck region, dilation of the external jugular vein on the affected side could be observed when the head is turned to the healthy side (Figure 5E) (14). In the absence of capillaries, the arterial blood directly flows into the venous system, resulting in the presence of the “steal phenomenon” (Figure 5B). Bleeding, ulceration, and infection caused by a “steal phenomenon”-induced ischemia of the skin and mucosa are common in the limbs, especially in the extremities (Figures 5B,C) (5). Nevertheless, normal color and texture of surface skin or mucosa might be seen in deep skin lesions (Supplementary Figure 2).

Type I simple pAVM patients were further divided into A and B subgroups. Type IA subgroup including patients whose AVM lesion confined to only one major anatomical region (MAR). In contrast, the lesions of pAVM patients from the Type IB subgroup involved at least two MARs. As Figures 5A–D shows, four of the patients' AVM lesions were relatively localized and were less diffuse than their counterparts (Figures 5E–H). All eight patients' CT images are presented in Supplementary Figure 3.

#### Type II simple pAVM (bone)

**Figure 6:**
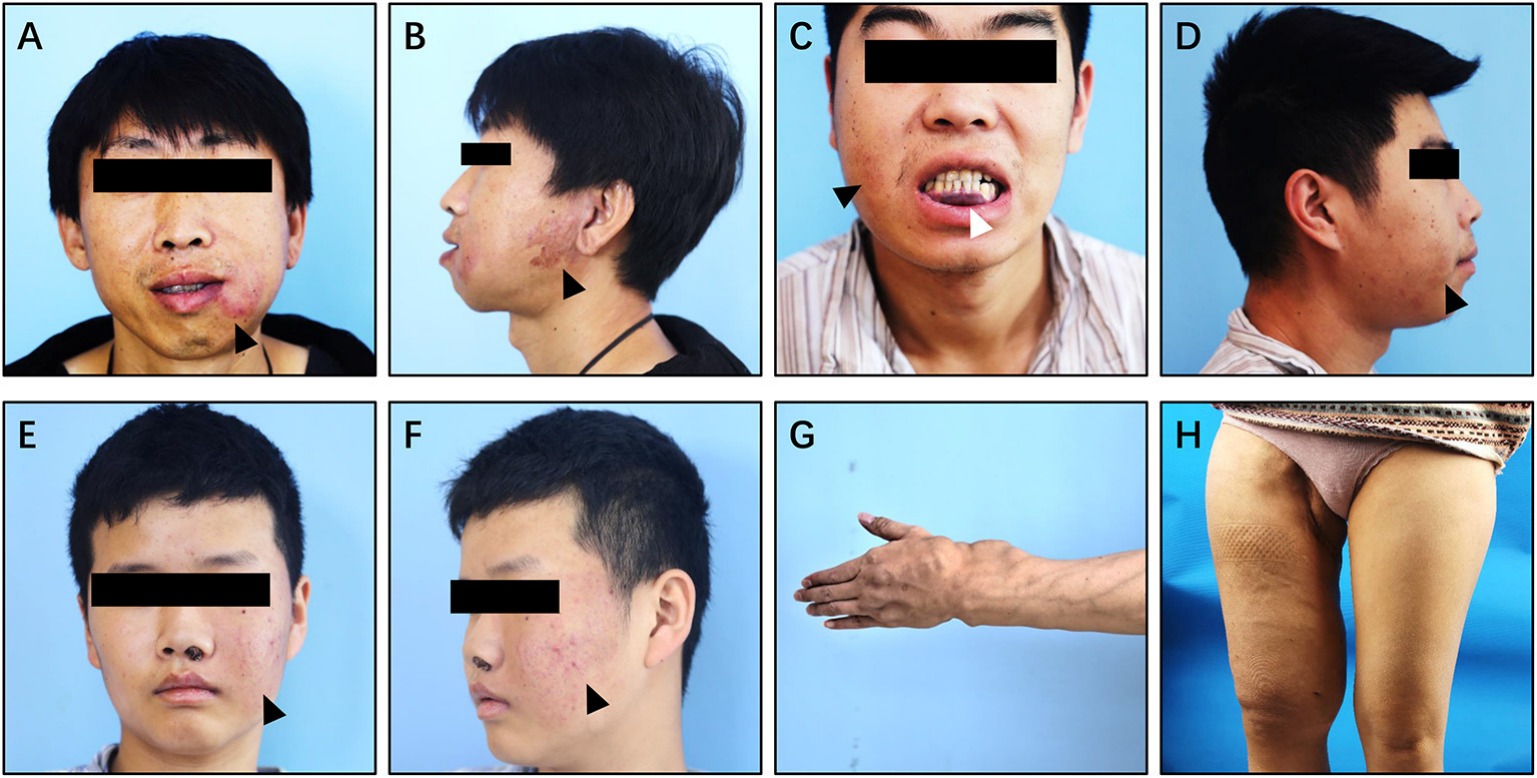
Clinical manifestation of Type II simple pAVM. **(A,B)** Patient 11 with mandibular AVM, cortex was intact (type IIA). **(C,D)** Patient 12 with mandibular AVM, cortex was broken (type IIB). **(E,F)** Patient 13 with maxillary AVM, cortex was intact (type IIA). **(G)** Patient 14 with AVM of left hand and forearm, cortex was broken (type IIB). **(H)** Patient 15 with AVM of right thigh, cortex was broken (type IIB). Black arrow: cutaneous erythema. White arrow: purple stain of the gingiva or oral mucosa.

Clinically, pAVM of the jaw mainly occurred in the molar or premolar region (Supplementary Figures 4A,D,G) (15). These lesions usually manifested as facial skin erythema or capillary dilation corresponding to lesions in the maxilla and mandible, accompanied by a rising skin temperature (Figures 6A–F; Supplementary Figures 6A,B,E,F). Root absorption is another possible manifestation of this condition (Supplementary Figure 4G). Due to the pressure on the inferior alveolar nerve, patients with mandibular pAVM can occasionally have numbness of the lower lip and/or submandibular region (1).

Type II simple pAVM patients always undergo recurrent, small amounts of spontaneous hemorrhage or even have episodes of uncontrolled acute hemorrhage (16). The acute hemorrhagic events mainly occurred during the children's tooth replacement period, which commonly begins at the age of 10, and maybe caused by tooth extraction and the replacement of deciduous teeth. Hemorrhage can also occur after the developmental phase of the bone (17). This evidence may account for the intriguing result of Supplementary Figure 5 that the majority of Type II simple pAVM patients came to receive treatments at the age of 11~20 years of age.

In addition to the head and neck region, pAVM can also occur in the long bones (Figures 6G,H). In addition to the common clinical manifestations, pAVM of the long bones can cause pathological fractures (18). The images of patients 11–15's are presented in Supplementary Figure 4.

#### Type III simple pAVM (viscus)

**Figure 7:**
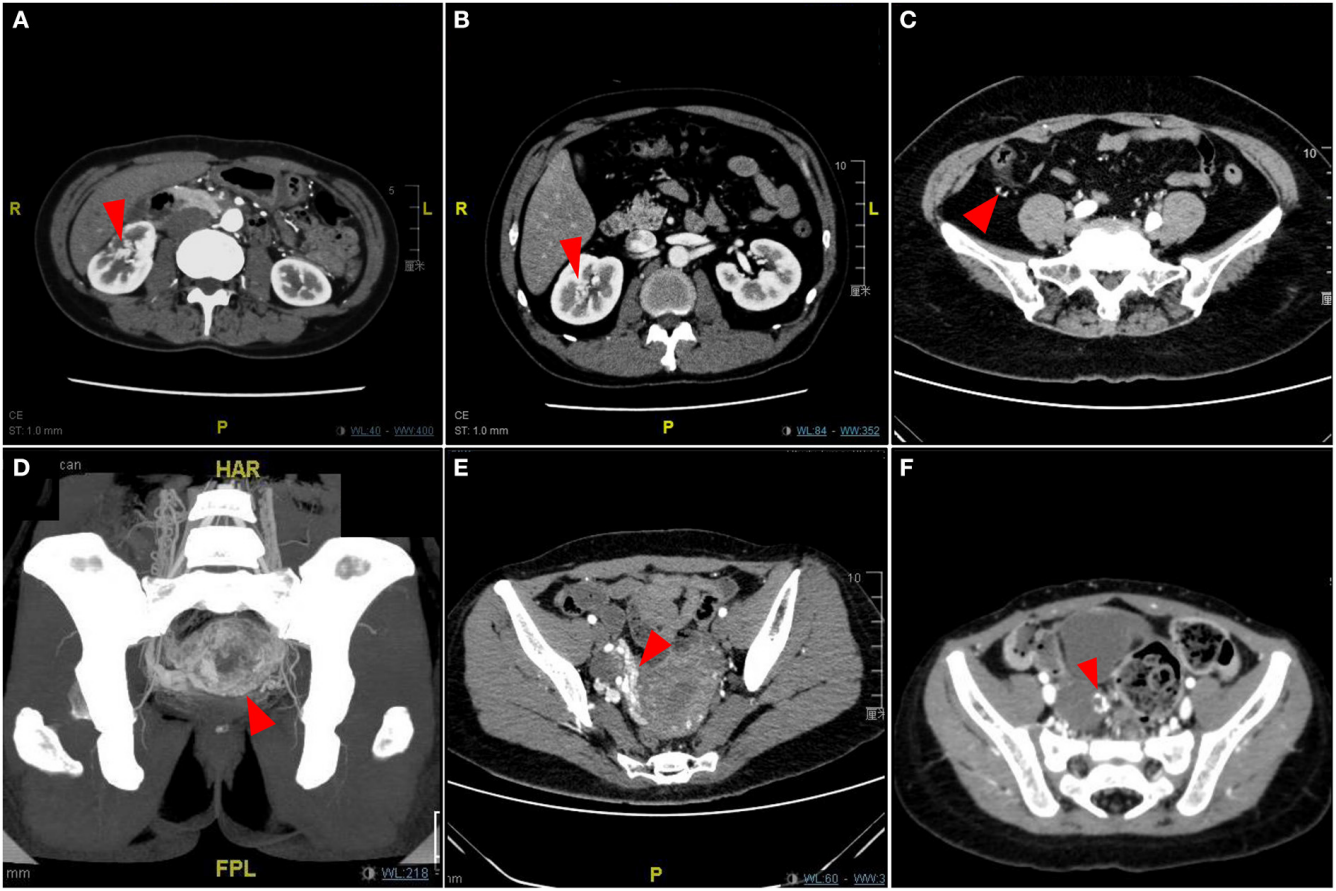
Imaging pictures of Type III simple pAVM patients. **(A)** Patient 18 with renal AVM. **(B)** Patient 19 with renal AVM. **(C)** Patient 20 with ascending colonic AVM. **(D)** Patient 21 with uterine AVM. **(E)** Patient 22 with uterine AVM. **(F)** Patient 23 with adnexal AVM. Red arrow: AVM lesion.

We classified the pAVM that primarily occurred in the visceral organs as Type III, which is an extremely rare kind of pAVM. The clinical symptoms of visceral AVMs are relatively insidious; some patients do not have any symptoms or only have symptoms that are difficult to distinguish and diagnose. The classic symptoms of renal AVMs include nephrogenic hypertension, pain, hematuria, abdominal bruits, and cardiopulmonary hyperload (Figures 7A,B) (19). Gastrointestinal AVMs can cause gastrointestinal bleeding, recurrent hematochezia, and even anemia, and sometimes the lesion may be misdiagnosed as hemorrhoids (Figure 7C) (20). Pelvic AVMs may present as asymptomatic lesions or present with symptoms such as abdominal pain, dysuria, frequent urination, hematuria, laboring dyspnea, or recurrent miscarriages (Figures 7D–F) (21). Given that the clinical manifestations of visceral AVMs are not as apparent as pAVM of the soft tissues and bone, imaging examinations are particularly necessary for the diagnosis and evaluation of these lesions.

#### Type IV simple pAVM (combined with cnsAVM)

**Figure 8:**
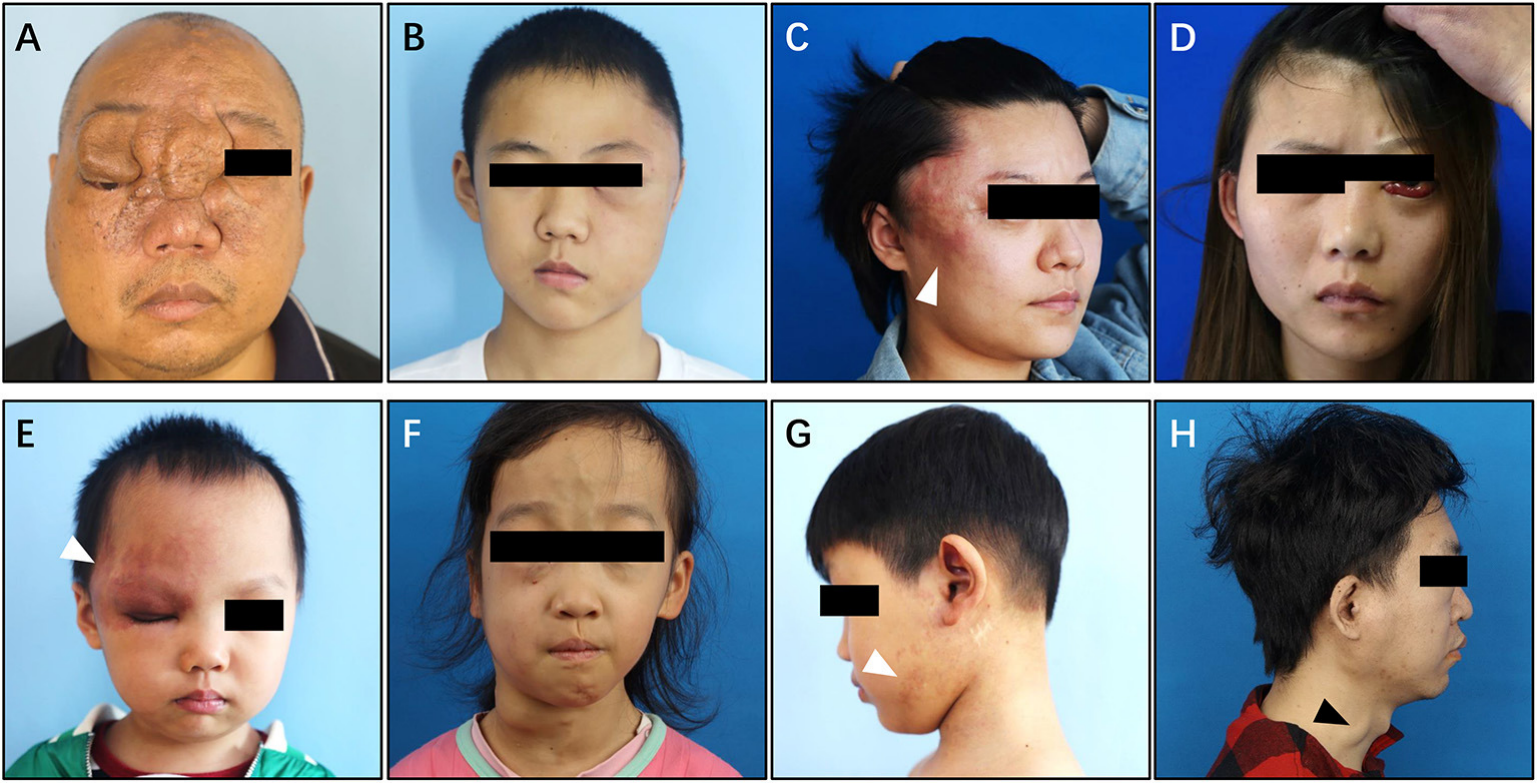
Clinical manifestation of Type IV simple pAVM. **(A)** Patient 24 with local soft tissue hyperplasia and exophthalmos of right eye. **(B)** Patient 25 with local swelling and exophthalmos of left eye. **(C)** Patient 26 with local swelling and pulsatile erythema. **(D)** Patient 27 with exophthalmos of left eye and conjunctival hyperplasia. **(E)** Patient 28 with local erythema and elevated cutaneous temperature. **(F)** Patient 29 with extensive swelling and dilated superficial veins, exophthalmos of left eye. **(G)** Patient 30 with sporadic erythema and elevated cutaneous temperature. **(H)** Patient 31 with sporadic swelling and dilated external jugular vein. White arrow: erythema. Black arrow: external jugular vein.

Among all of the 1,070 simple pAVM patients, 11 patients also had AVM lesions in the central nervous system (Figure 8). All of these patients' CT images were presented in Supplementary Figures 7, 8.

In addition to the typical skin manifestations that are associated with Type I simple pAVM (Figure 8; Supplementary Figures 7D,G), Type IV simple pAVM patients sometimes present with clinical symptoms of cnsAVMs, such as headache, epilepsy, intracranial bruit, exophthalmos, and even subarachnoid hemorrhage, intracranial hemorrhage or subdural hemorrhage (22). Among these patients, patients 24, 27, 29 presented with exophthalmos of the affected side (Figures 8A,D,F). Patient 34 with a lesion involving the spinal cord, whose complaint was numbness of the lower limbs, and severe scoliosis could be observed (Supplementary Figures 8I–L).

## Discussion

Given the rarity of simple pAVM, there have not been enough large-scale studies previously, especially in studies that have used statistical analyses focusing on the epidemiology of simple pAVM. In the present study, we shared the 5 year experience derived from the sample size of over 1,000 patients.

According to the sex distribution of our cohort, simple pAVM was more common in males than females. We further consolidated the view that sex difference could be regarded as the influence factor of simple pAVM occurrence by the chi-squared test. These results proved and refined the conclusions of the previous report (3). Another intriguing phenomenon was the special age distribution of simple pAVM patients. AVM is a series of congenital defects that already exist when the patients are born; nevertheless, due to its insidious progression, patients and their families do not notice symptoms of this disease until the lesion evolves to a relatively severe status.

On account of the complex manifestations of simple pAVM in bone, differential diagnoses should be performed in a sophisticated way. For example, Type IIA simple pAVM may not present with cutaneous pulsation, while cutaneous pulsation can always be felt in Type IIB patients. Patients with maxillary AVM may undergo recurrent epistaxis on the affected side due to telangiectasia of the nasal mucosa (Figures 6E,F). For Type IIB simple pAVM, an oral examination may show red or purple staining of the gingiva or oral mucosa, which may be caused by the dilatation of superficial capillaries or the local outflow veins (Figure 6C). In contrast to jaws, Type IIA simple pAVM of the long bones are always insidious, which means that there are no obvious manifestations on the skin or sensory signals until the nidus breaks through the cortex (Figure 6G; Supplementary Figures 4J–M). However, for Type IIB simple pAVM in the lower limbs, in the presence of relatively stout muscles, the cutaneous manifestations may not be obvious because there may be only local swelling (Figure 6H; Supplementary Figures 4N–R). Therefore, imaging examinations are required to make a definite diagnosis.

Additionally, the differentiation between Type I and Type IIB is also pivotal in the diagnosis of simple pAVM. Owing to the similarity of the cutaneous manifestations, including erythema, elevated skin temperature, pulsation, and obvious dilated outflow veins, clinicians are prone to overlook intraosseous lesions; thus, imaging is the gold standard to confirm the subtype. Patient 2, 3, 6, 7, and Patient 14, 15 were the typical pairs to demonstrate the differences: although the lesion in the soft tissue was adjacent to the bone, the cortex was continuous and was not involved, and there were no highlighted areas seen in the marrow cavity (Supplementary Figures 3B,C,F,G). In contrast, a discontinuous or perforated cortex and highlighted areas in the marrow cavity could be seen in Supplementary Figures 4K,L,N–Q.

Another highlight of the present study cohort was a series of simple pAVM patients who suffered from cnsAVM simultaneously. Despite the uncertainty of the origin, previous studies on the molecular genetic pathogenesis of AVM might shed light on the etiology of type IV simple pAVM. Nikolaev et al. (23) proposed that the activating mutation of *KRAS* (Kirsten rat sarcoma viral oncogene homolog)^*G*12*V*^ in brain-AVM gave rise to the activation of *ERK* (extracellular signal-regulated kinase), increased expression of genes related to angiogenesis and Notch signaling, and enhanced migratory behavior. Schmidt et al. (24) provided evidence that *KRAS*^*G*12*D*^, a mosaic *KRAS* variant, was active in pAVM patient lesions, which is in contrast to normal tissue, and similar findings were proven by other studies performed both *in vitro* and *in vivo* (25, 26). Thus, we postulated that the *KRAS* mutation was likely to be the key factor leading to pAVM patients' acquired CNS lesions or vice versa. Although more work is needed soon, vascular malformation-related genetic screening has been carried out in our center to obtain a far-reaching understanding of the gene mutation-induced pathogenic mechanisms and the protocols for targeted therapy in simple pAVM patients. Nevertheless, in consideration of the particularity of these patients, it is difficult to identify whether the peripheral lesions or the CNS lesions are primary, and we temporarily classified them as Type IV simple pAVM. Undoubtedly, close follow-up and further investigation are needed in future studies.

To date, specialists in vascular anomalies have been dedicated not only to improving the strategies for treatments or hospitalization but also to finding a more reasonable and effective way to classify the vascular anomalies to obtain an in-depth understanding of the vascular anomalies and the recommendations for differential diagnosis (27).

There have been several classifications for vascular anomalies. First, the Hamburg classification distinguishes each type of vascular anomaly from the perspective of embryology (28). AVMs were divided into two subtypes: truncular and extratruncular forms. Extratruncular (ET) forms develop in the earlier stages of embryogenesis, and they possess proliferative embryonic potential when stimulated (e.g., trauma, surgery, hormonal changes) due to the presence of mesenchymal cells (angioblasts) (29). Truncular (T) forms develop in the later stages of embryogenesis and lack embryonic characteristics, but they have a more severe hemodynamic impact (30). Additionally, ISSVA Classification divided the vascular anomalies based on the Hamburg Classification and lesion flow characteristics into fast-flow and slow-flow vascular malformations (9).

For classifications exclusive to AVM, Houdart E, as well as Yakes et al. (7, 31) classified AVM lesions located in the central nervous system and peripheral body based on the arteriographic morphology of the “nidus” (32), respectively. These classification systems refined the previous editions and provided references for clinical treatment. However, due to the operative and invasive nature of angiography, AVM classifications mentioned above only focused on the angiographic results, imposed restrictions on the overall judgment of AVM (both primary lesion and surrounding tissue or structure). In light of the still poor understanding of AVM, misdiagnoses were widely reported (33–35). Thus, the correct pre-operation diagnosis of AVM not only has a guiding effect on therapeutic strategies but determines the prognosis of the patient to a large extent. The authors from Birmingham proposed the classification for venous malformations (VMs) based on the extent of tissue involvement (36), which reminded us that proposing a classification depicting AVM from the perspective of range and nature of the primary lesion is of significance. Furthermore, Puig et al. (37) once classified VMs according to the angio-architectural features, promoting the management of VM. Compared with VM, AVM [although with a lower incidence than VM (38)] was multitudinous and more aggressive. In the present work, the anatomical classification for simple pAVM was expected to make the diagnosis of pAVM more organized.

On the macro level, we have received and treated pAVM patients from every province of China. Despite the large sample size, the number of patients from relatively remote areas was sufficient either because of the long-distance or due to the scarcity of cognition of pAVM, indicating the inadequate statistical capability when evaluating a population from a single center. Thus, multicenter cooperation should be conducted in future studies to cover the disadvantages.

## Data availability statement

The raw data supporting the conclusions of this article will be made available by the authors, without undue reservation.

## Ethics statement

This study was approved by the Human Research Ethics Committee of the Ninth People's Hospital, Shanghai Jiao Tong University School of Medicine (Shanghai, China). Given the retrospective nature of this study, the need for informed consent was waived.

## Author contributions

DW: conceptualization, methodology, and writing-review and editing. YS: formal analysis, investigation, and roles/writing-original draft. ZW: data curation. XF: funding acquisition. LS: project administration. JW, JY, and CJ: supervision. LZhe, XY, and MW: validation. YH, XL, and LZha: visualization. All authors contributed to the article and approved the submitted version.

## Funding

This study was funded by the National Natural Science Foundation of China (No. 81871458), the Health Clinical Research Project of Shanghai Municipal Health Commission (No. 202040328), Fundamental research program funding of Ninth People's Hospital affiliated to Shanghai Jiao Tong University School of Medicine (No. JYZZ076), and Clinical Research Program of 9th People's Hospital affiliated to Shanghai Jiao Tong University School of Medicine (Nos. JYLJ201801, JYLJ201911, and JYLJ202111).

## Conflict of interest

The authors declare that the research was conducted in the absence of any commercial or financial relationships that could be construed as a potential conflict of interest.

## Publisher's note

All claims expressed in this article are solely those of the authors and do not necessarily represent those of their affiliated organizations, or those of the publisher, the editors and the reviewers. Any product that may be evaluated in this article, or claim that may be made by its manufacturer, is not guaranteed or endorsed by the publisher.

## Supplementary material

Supplementary Figure 1 Detailed number of simple pAVM in each province of China.

Supplementary Figure 2 Type I simple pAVM patients with deep lesions. **(A)** Normal skin manifestation could be observed in the patient 9 with deep AVM of left thigh. **(B–D)** MRI images of the AVM lesion. **(E)** Normal skin manifestation could be observed in patient 10 with deep AVM of left thigh. **(F–H)** MRI images of the AVM lesion. Red arrow: representative “flowing void effect.”

Supplementary Figure 3 Imaging pictures of Type I simple pAVM patients. **(A–H)** Patient 1-8's images of enhanced-contrast CT. Red arrow: AVM lesion. Yellow arrow: Dilated external jugular vein.

Supplementary Figure 4 Imaging pictures of Type II simple pAVM patients. **(A)** Oral pantomography of patient 11. **(B,C)** Enhanced-contrast CT of patient 11. **(D)** Oral pantomography of patient 12. **(E,F)** Enhanced-contrast CT of patient 12. **(G)** Oral pantomography of patient 13. **(H,I)** Enhanced-contrast CT of patient 13. **(J–M)** Enhanced-contrast CT of patient 14. **(N–R)** Enhanced-contrast CT of patient 15. Red dotted box: macrocystic low-density shadow on oral pantomography indicating intact cortex. Red dotted ellipse: discontinuous cortex. Microcystic low-density shadow as well as “soap bubble” or “honey comb” on oral pantomography. White arrow: root absorption. Red arrow: highlighted images of marrow cavity. Yellow arrow: Dilated mandibular canal.

Supplementary Figure 5 Number of patients with type II simple pAVM of each age group (*P* = 0.04).

Supplementary Figure 6 Clinical manifestation of Type II simple pAVM. **(A–D)** Patient 16 with maxillary AVM, cortex was intact (IIA). **(E–H)** Patient 17 with mandibular AVM, cortex was intact (IIA). Dark arrow: cutaneous erythema. Red arrow: highlighted images of marrow cavity (AVM lesions).

Supplementary Figure 7 Imaging pictures of Type IV simple pAVM patients. **(A–C)** Enhanced-contrast CT images of patient 24. **(D)** Lateral picture of patient 25. **(E,F)** Enhanced-contrast CT images of patient 25. **(G)** Anterior picture of patient 26. **(H,I)** Enhanced-contrast CT images of patient 26. **(J–L)** Enhanced-contrast CT images of patient 27. **(M–O)** Enhanced-contrast CT images of patient 28. **(P–R)** Enhanced-contrast CT images of patient 29. **(S–U)** Enhanced-contrast CT images of patient 30. **(V–X)** Enhanced-contrast CT images of patient 31. Red arrow: intracranial lesion. Yellow arrow: exophthalmos. Dark arrow: dilated external jugular vein. Red dotted circle: the “steal phenomenon.”

Supplementary Figure 8 Imaging pictures of Type IV simple pAVM patients. **(A)** Posterior picture of patient 32. **(B–D)** Enhanced-contrast CT images of patient 32. **(E)** Lateral picture of patient 33. **(F–H)** Enhanced-contrast CT images of patient 33. **(I)** Posterior picture of patient 34. **(J–L)** Enhanced-contrast CT images of patient 34. Red arrow: intracranial lesion.
