# Supplementary figures and images for: Novel classification for simple peripheral arteriovenous malformations based on anatomic localization: Prevalence data from the tertiary referral center in China
Source: Front Cardiovasc Med. 2022 Jul 22;9:935313. doi: 10.3389/fcvm.2022.935313 (PMC9356220; doi:10.3389/fcvm.2022.935313)

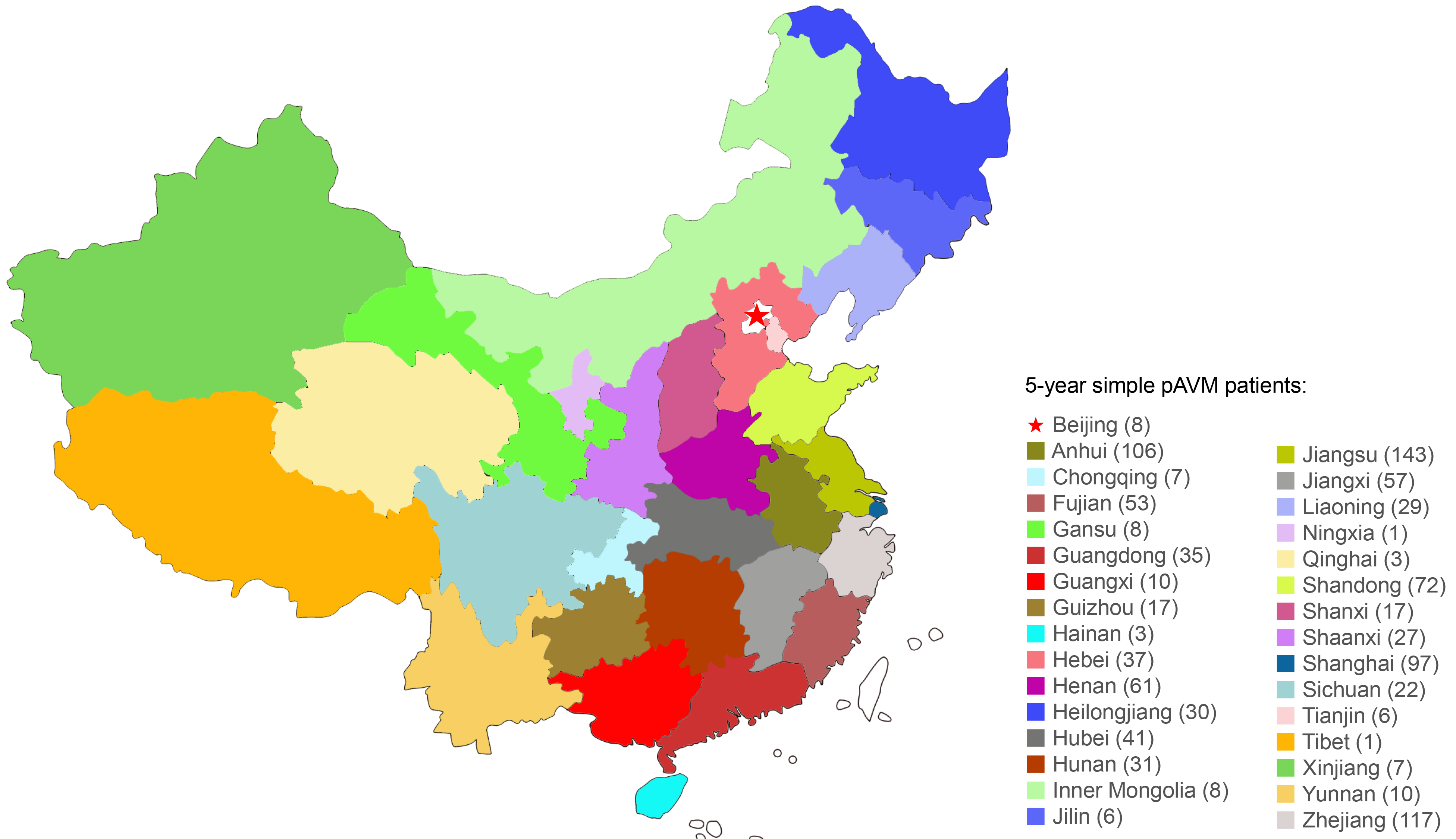

Supplement: Supplementary Figure 1 — Detailed number of simple pAVM in each province of China. [file Image_1.TIF]

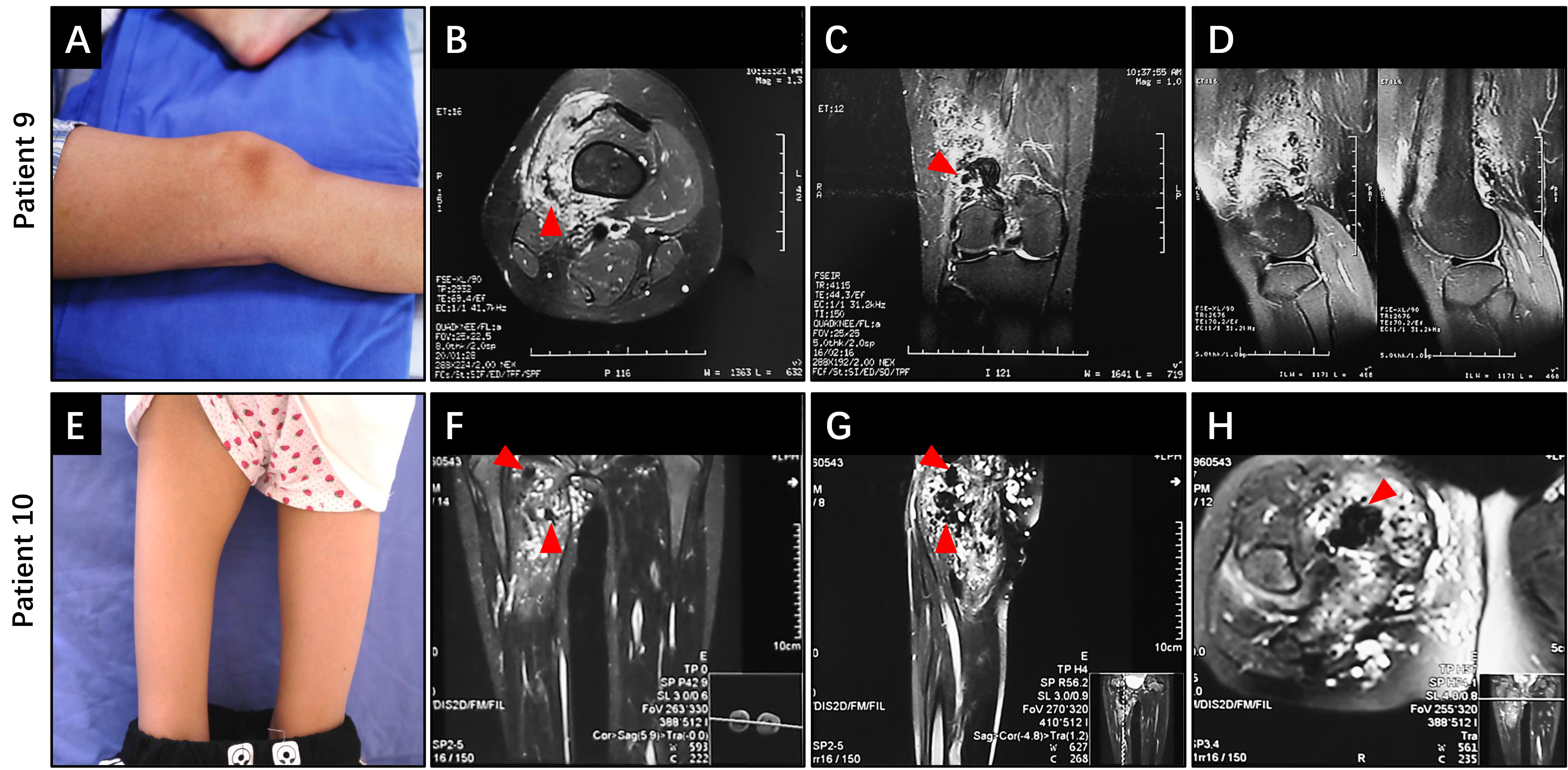

Supplement: Supplementary Figure 2 — Type I simple pAVM patients with deep lesions. (A) Normal skin manifestation could be observed in the patient 9 with deep AVM of left thigh. (B–D) MRI images of the AVM lesion. (E) Normal skin manifestation could be observed in patient 10 with deep AVM of left thigh. (F–H) MRI images of the AVM lesion. Red arrow: representative “flowing void effect.” [file Image_2.TIF]

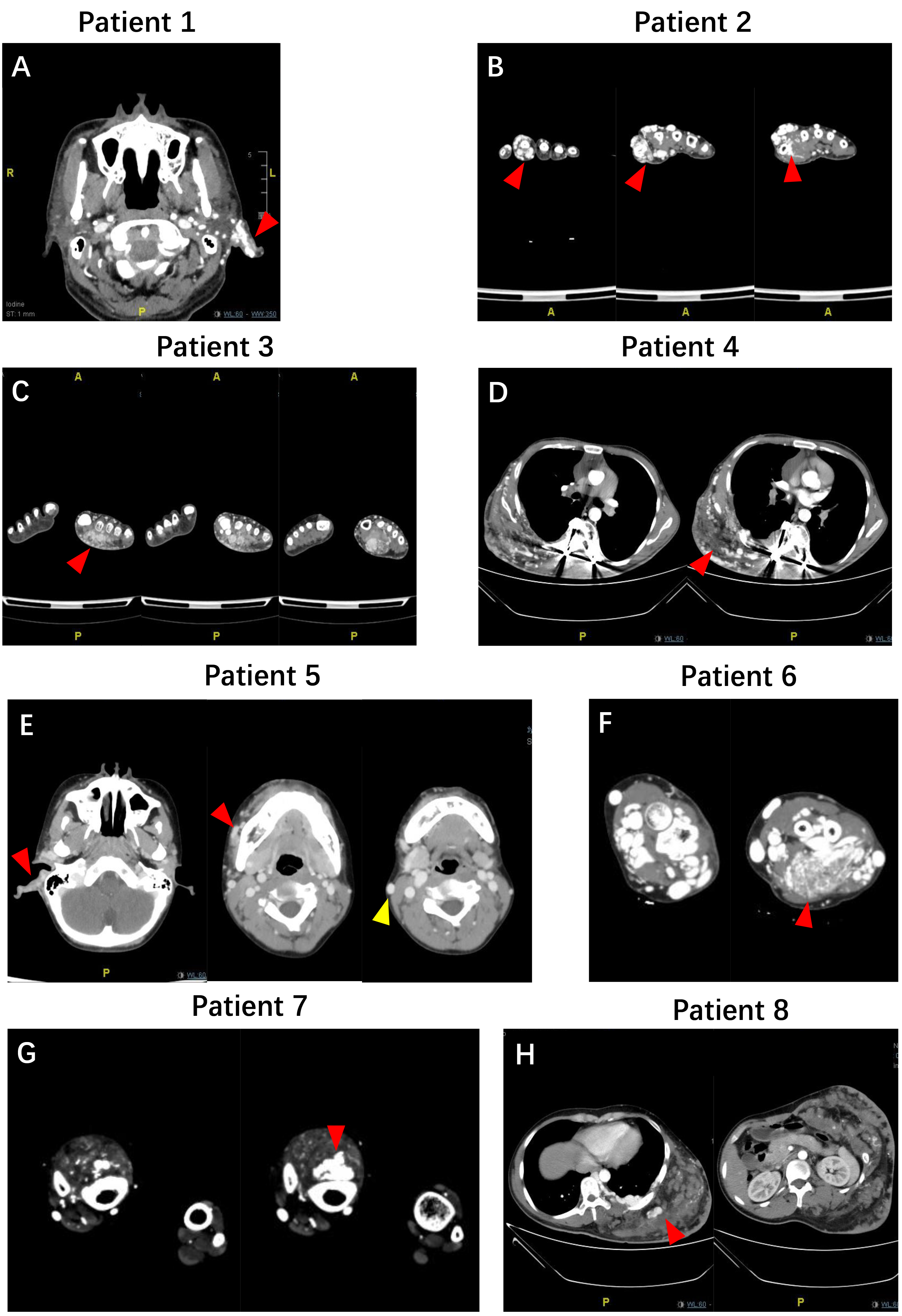

Supplement: Supplementary Figure 3 — Imaging pictures of Type I simple pAVM patients. (A–H) Patient 1-8's images of enhanced-contrast CT. Red arrow: AVM lesion. Yellow arrow: Dilated external jugular vein. [file Image_3.TIF]

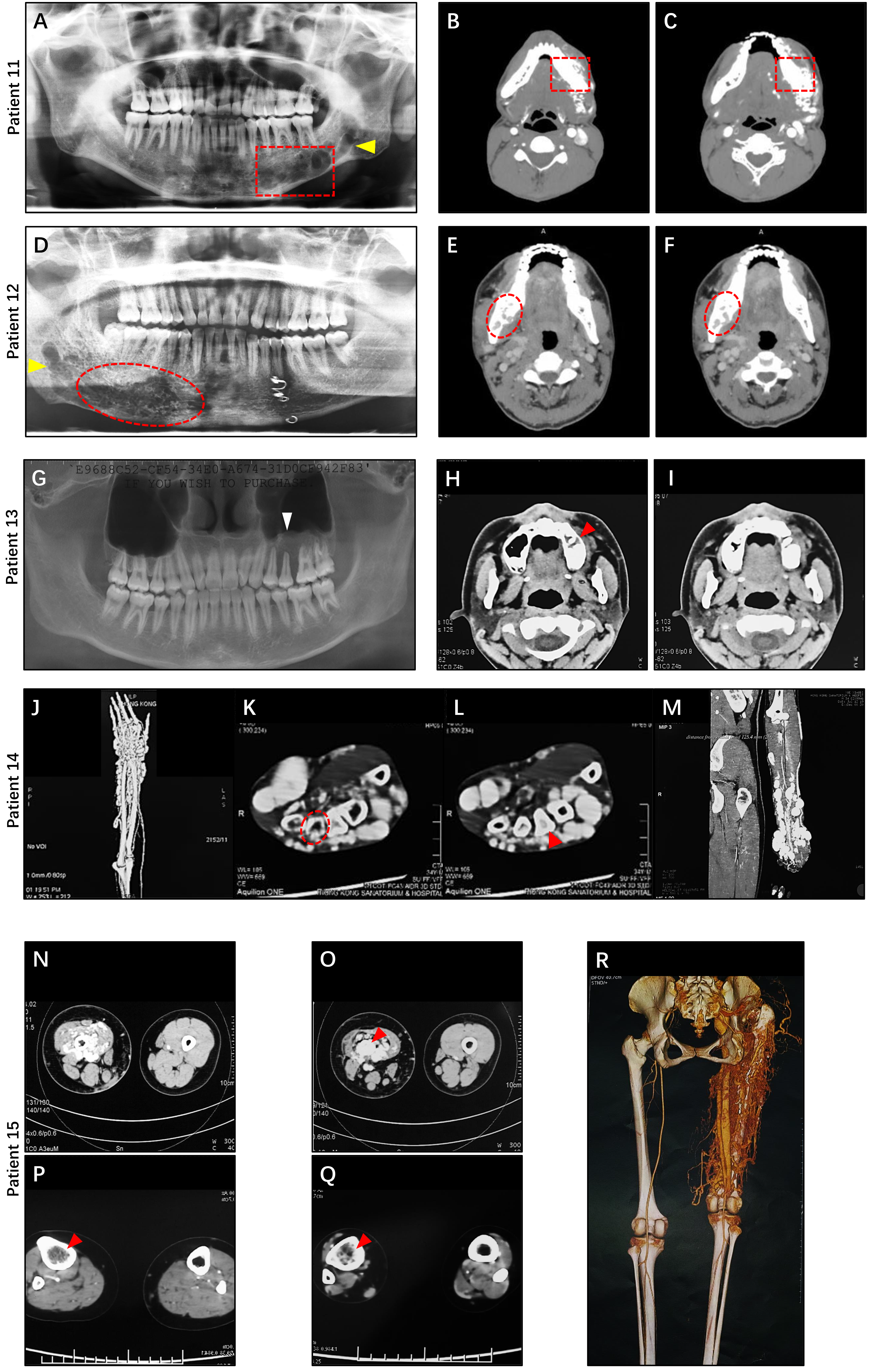

Supplement: Supplementary Figure 4 — Imaging pictures of Type II simple pAVM patients. (A) Oral pantomography of patient 11. (B,C) Enhanced-contrast CT of patient 11. (D) Oral pantomography of patient 12. (E,F) Enhanced-contrast CT of patient 12. (G) Oral pantomography of patient 13. (H,I) Enhanced-contrast CT of patient 13. (J–M) Enhanced-contrast CT of patient 14. (N–R) Enhanced-contrast CT of patient 15. Red dotted box: macrocystic low-density shadow on oral pantomography indicating intact cortex. Red dotted ellipse: discontinuous cortex. Microcystic low-density shadow as well as “soap bubble” or “honey comb” on oral pantomography. White arrow: root absorption. Red arrow: highlighted images of marrow cavity. Yellow arrow: Dilated mandibular canal. [file Image_4.TIF]

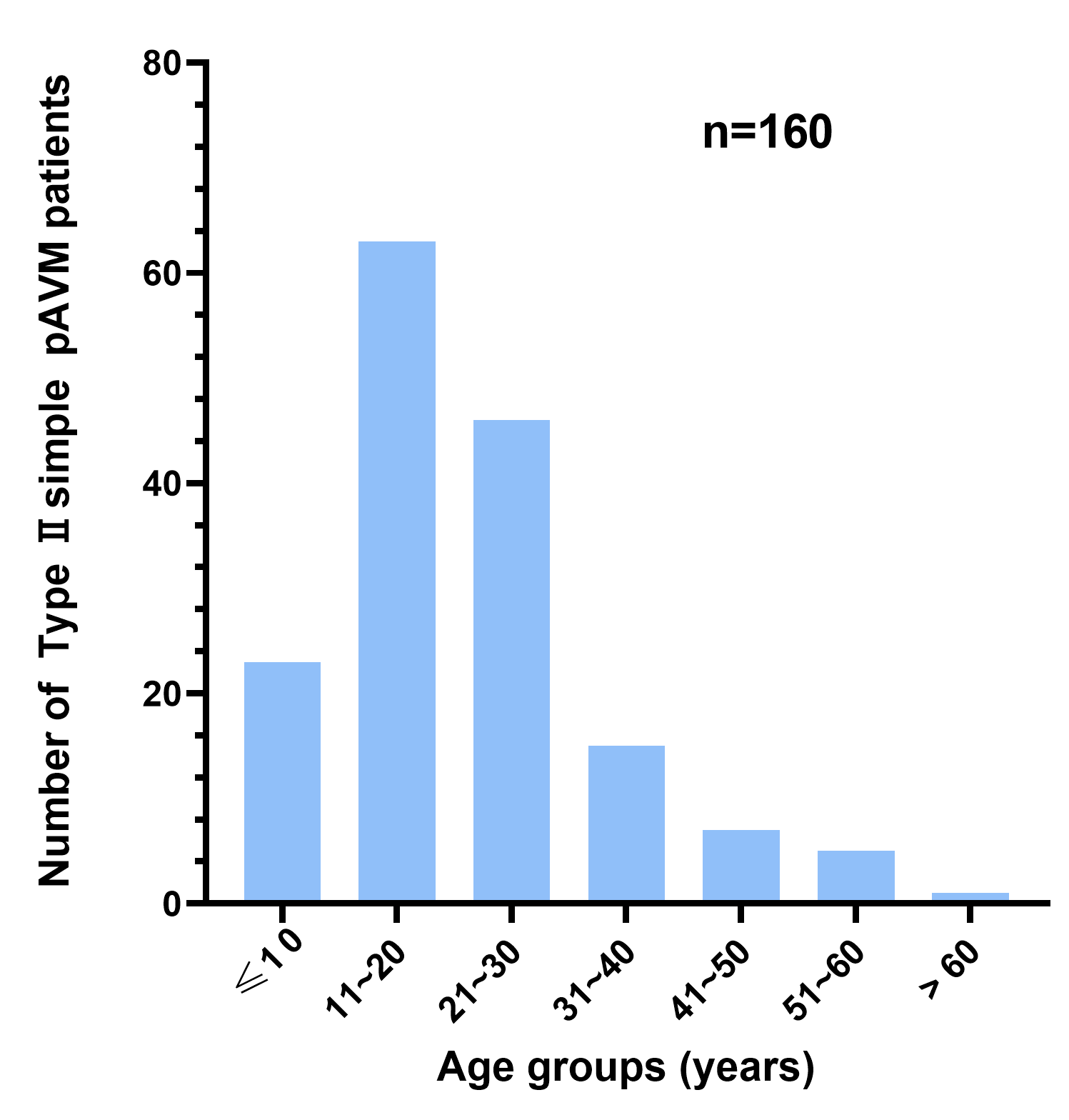

Supplement: Supplementary Figure 5 — Number of patients with type II simple pAVM of each age group (P = 0.04). [file Image_5.TIF]

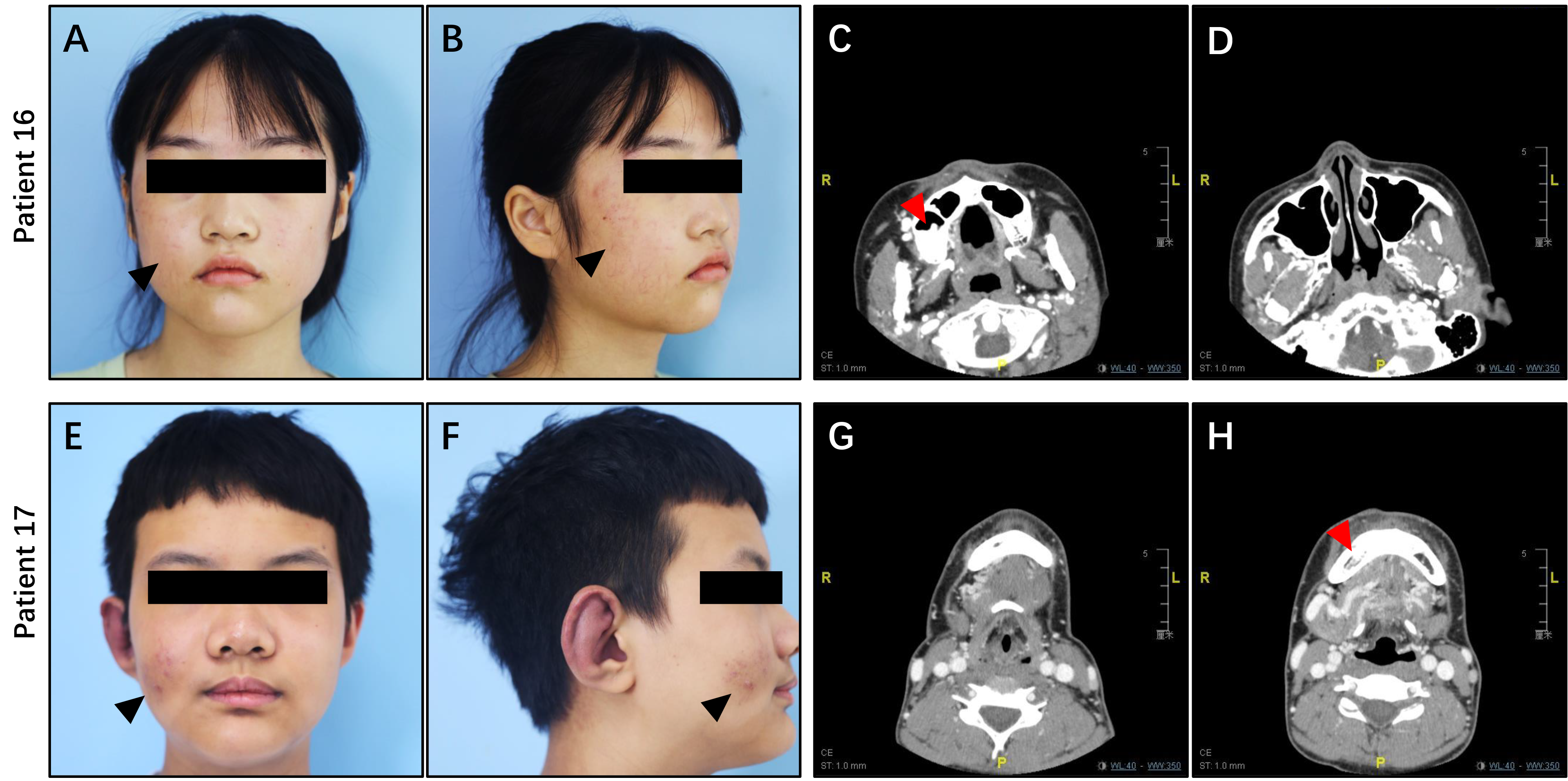

Supplement: Supplementary Figure 6 — Clinical manifestation of Type II simple pAVM. (A–D) Patient 16 with maxillary AVM, cortex was intact (IIA). (E–H) Patient 17 with mandibular AVM, cortex was intact (IIA). Dark arrow: cutaneous erythema. Red arrow: highlighted images of marrow cavity (AVM lesions). [file Image_6.TIF]

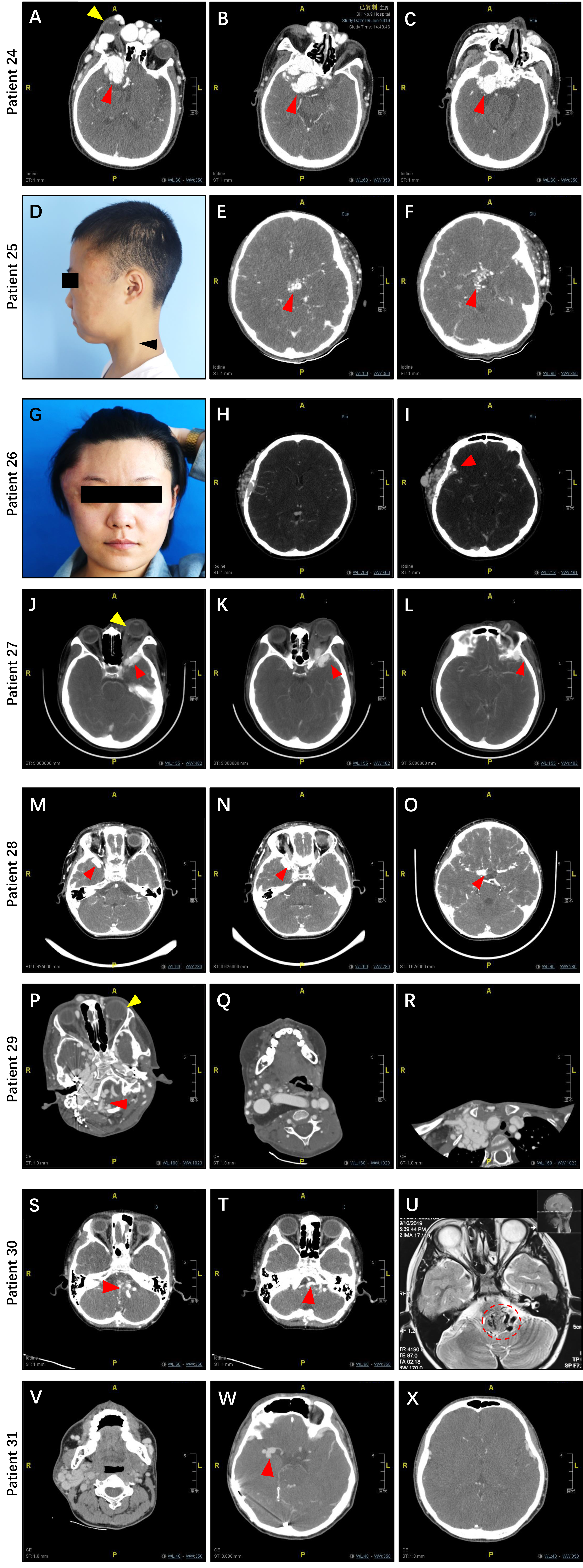

Supplement: Supplementary Figure 7 — Imaging pictures of Type IV simple pAVM patients. (A–C) Enhanced-contrast CT images of patient 24. (D) Lateral picture of patient 25. (E,F) Enhanced-contrast CT images of patient 25. (G) Anterior picture of patient 26. (H,I) Enhanced-contrast CT images of patient 26. (J–L) Enhanced-contrast CT images of patient 27. (M–O) Enhanced-contrast CT images of patient 28. (P–R) Enhanced-contrast CT images of patient 29. (S–U) Enhanced-contrast CT images of patient 30. (V–X) Enhanced-contrast CT images of patient 31. Red arrow: intracranial lesion. Yellow arrow: exophthalmos. Dark arrow: dilated external jugular vein. Red dotted circle: the “steal phenomenon.” [file Image_7.TIF]

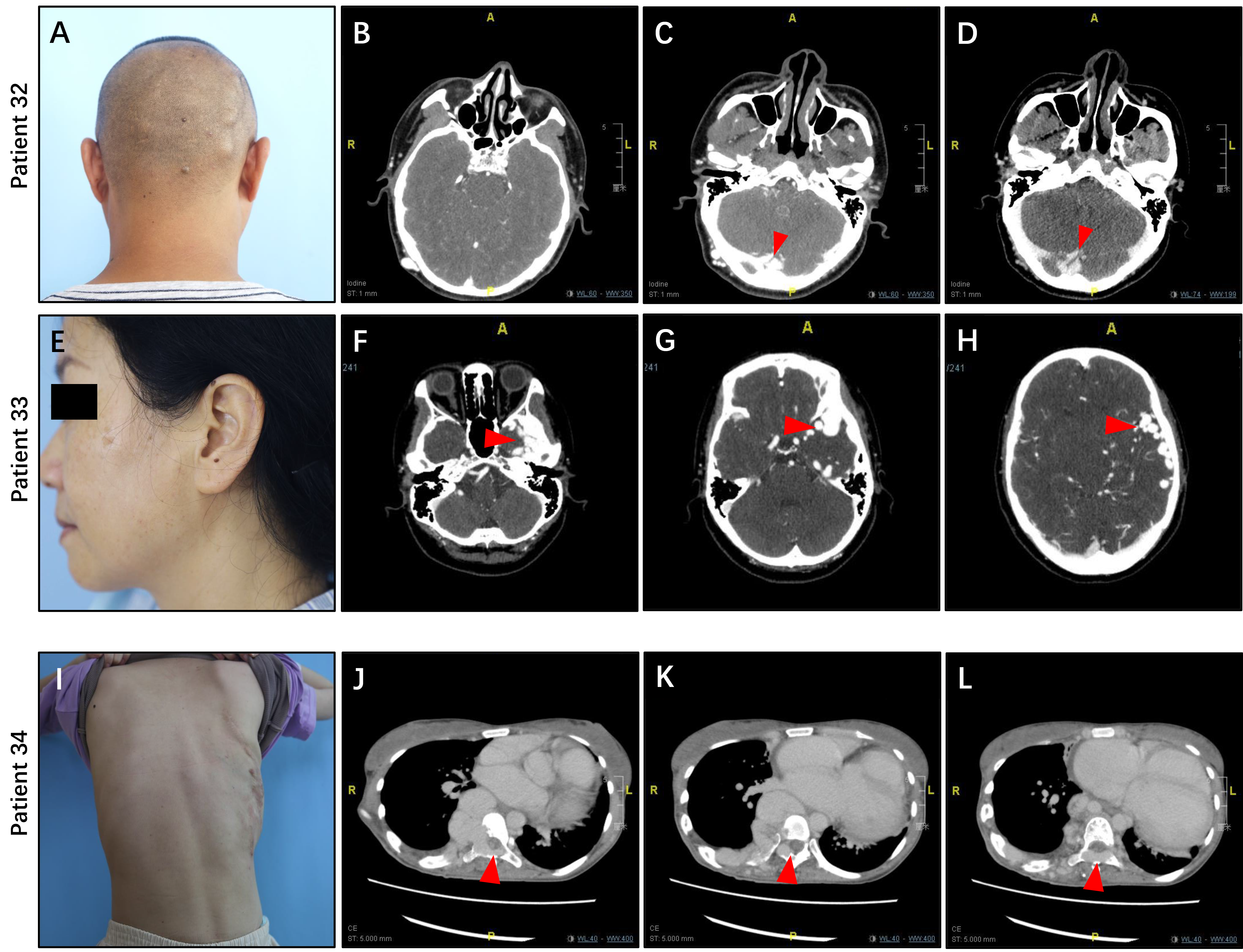

Supplement: Supplementary Figure 8 — Imaging pictures of Type IV simple pAVM patients. (A) Posterior picture of patient 32. (B–D) Enhanced-contrast CT images of patient 32. (E) Lateral picture of patient 33. (F–H) Enhanced-contrast CT images of patient 33. (I) Posterior picture of patient 34. (J–L) Enhanced-contrast CT images of patient 34. Red arrow: intracranial lesion. [file Image_8.TIF]
